# Exploring the Effects of Pharmacological, Psychosocial, and Alternative/Complementary Interventions in Children and Adolescents With Attention-Deficit/Hyperactivity Disorder: Meta-Regression Approach

**DOI:** 10.1093/ijnp/pyab034

**Published:** 2021-06-04

**Authors:** Kung-Han Yang, Hsien-Yuan Lane, Yue-Cune Chang, Ruu-Fen Tzang

**Affiliations:** 1 Department of Applied Mathematics, Chinese Culture University, Taipei, Taiwan; 2 Department of Mathematics, Tamkang University, Taipei, Taiwan; 3 Department of Psychiatry, Mackay Memorial Hospital, Taipei, Taiwan; 4 Mackay Junior College of Medicine, Nursing, and Management, Taipei, Taiwan; 5 Department of Psychiatry, China Medical University Hospital, Taichung, Taiwan; 6 Graduate Institute of Biomedical Sciences, China Medical University Medical College, Taichung, Taiwan; 7 Department of Psychology, College of Medical and Health Sciences, Asia University, Taichung, Taiwan

**Keywords:** ADHD, behavior therapy, meta-regression, pharmacotherapy, treatment efficacy

## Abstract

**Background:**

There have been various therapies for attention-deficit/hyperactivity disorder (ADHD), but the previous meta-analysis of ADHD efficacy remains unclear. This study aims to systemically meta-regress the effect sizes (ES) of psychostimulant pharmacotherapy (methylphenidate and lisdexamfetamine), non-stimulant pharmacotherapy (atomoxetine and alpha-2 agonists), psychosocial therapy (parental behavioral therapy [PBT]), combination therapy (psychostimulant plus PBT), and alternative/complementary interventions to determine the right treatment for ADHD.

**Methods:**

We searched various ADHD interventions from the MEDLINE and PubMed databases (National Center for Biotechnology Information) between January 1, 1980, and July 30, 2018. Following the meta-analysis of random effects, the meta-regression analyses were used to explore factors potentially influencing treatment efficacy. The confounding variables included type of treatment, type of study, age, type of symptom scale used, and year of publication.

**Results:**

A total of 107 trials (n = 9883 participants) were included. After adjustment, compared with the psychostimulant therapy (28 trial, 2134 participants), non-stimulant pharmacotherapy (28 trials, 4991 participants) and alternative/complement intervention (25 trials, 1195 participants) were less effective by the ES of −0.384 (*P* = .004) and −0.419 (*P* = .028), respectively. However, compared with psychostimulant, PBT (19 trials, 1122 participants; ES = −0.308, *P* = .095) and the combination of psychostimulant and PBT (7 trials, 441participants; ES = −0.196, *P* = .209) did not differ significantly.

**Conclusions:**

Psychostimulant therapy surpassed non-stimulant pharmacotherapy and alternative/complement intervention. Psychostimulant therapy, PBT, and the combination of psychostimulant therapy and PBT appear to be similar in efficacy according to this meta-regression.

**Significance Statement**

Attention-deficit hyperactivity disorder (ADHD) is a common neurodevelopmental disorder among children and adolescents. The right choice of ADHD treatment is always a major concern of parents. However, previous network meta-analysis of ADHD efficacy by indirectly estimating effect size remains unclear in comparing the effects of pharmacotherapy, psychosocial intervention, and alternative/complementary approaches. Understanding the treatment efficacy of ADHD by new meta-regression analysis is an effective and exploratory analyses for heterogeneity according to the studies of cross-level interactions. This meta-regression is eligible in directly comparing the effects of ADHD treatments by systemically reviewing the “interaction analysis” of pharmacotherapy (psychostimulant and non-stimulant pharmacotherapy), psychosocial intervention, and alternative/complementary approaches. In conclusion, this meta-regression indicates that a combination therapy of psychopharmacotherapy and parental behavioral therapy provides an evidence-based ADHD treatment model.

## Introduction

Attention-deficit/hyperactivity disorder (ADHD) is a common neurodevelopmental disorder (Polanczyk et al., 2007; Xu et al., 2018). The possible consequences of inadequate treatment of ADHD include antisocial personality disorders, substance-related and addictive disorders (Yoshimasu, 2016), internet addiction (Seyrek et al., 2017), and depression (Biederman et al., 2008; Knouse et al., 2013). There are diverse ADHD treatments, such as psychostimulant pharmacotherapy (methylphenidate [MPH] and lisdexamfetamine), non-stimulant pharmacotherapy (atomoxetine [ATX] and alpha-2 agonists such as clonidine and guanfacine), psychosocial therapy (parental behavioral therapy [PBT]), combination therapy (psychostimulant plus PBT), and alternative/complementary interventions.

In developed countries, the first choice of ADHD treatment is pharmacotherapy, as indicated by ADHD treatment guidance from the UK National Institute for Health and Care Excellence (Hall et al., 2016), the British Association for Psychopharmacology guidelines in the United Kingdom (Nutt et al., 2007), and Multimodal Treatment Study of Children With ADHD trial results in the United States (Wolraich et al., 2011). In some developing countries, PBT and alternative/complementary therapies are prevalent. A more updated meta-analysis methodology is needed to elucidate the effective treatment of ADHD.

MPH has been reported to be effective for enhancing cognition (Coghill et al., 2014) and reducing ADHD symptoms (Reichow et al., 2013; Kambeitz et al., 2014), with an effect size (ES) of 0.8–1.0 (Banaschewski et al., 2008; Faraone and Buitelaar, 2010). Moreover, the treatment effect of MPH sometimes can last for a longer time (Maia et al., 2017).

Non-stimulant pharmacotherapy, ATX, also improves overall ADHD symptoms (Michelson et al., 2001; Kelsey et al., 2004; Cunill et al., 2013; Asherson et al., 2014; Handen et al., 2015) and secondary outcomes (Schwartz and Correll, 2014). The mean ES of ATX was 0.64 (Bloch, 2014). ATX also relieves symptoms of oppositional defiant disorder in children with ADHD (Bangs et al., 2008; Dittmann et al., 2011; Asherson et al., 2015). Alpha-2 agonists can also treat ADHD (Hirota et al., 2014), with a moderate ES of 0.58 (Cinnamon Bidwell et al., 2010). The result of a meta-analysis and meta-regression of 87 randomized controlled clinical trials of ADHD treatment showed that the efficacy of pharmacological treatment for ADHD remained stable over time (Castells et al., 2020). MPH appeared more effective than non-stimulant pharmacotherapy (Padilha et al., 2018).

ADHD treatment guidelines established by The Multisite Multimodal Treatment Study of Children with ADHD (Group, 1999; Wolraich et al., 2011) and the American Academy of Pediatrics (Briars and Todd, 2016) suggest that pharmacotherapy plus PBT is the most effective ADHD treatment (Atkinson and Hollis, 2010; Wolraich, 2012; Golubchik et al., 2018). PBT, aiming to shape the behavior of children, reduce parental stress, and enhance parental confidence (Wang et al., 2014; Huang et al., 2015; Lange et al., 2016; Mohammadi et al., 2016), has been demonstrated to be effective in some studies (Charach et al., 2013; Mulqueen et al., 2015) but not in others (Jadad et al., 1999; Brown et al., 2005; Zwi et al., 2011; De Crescenzo et al., 2017). A previous meta-analysis found that parenting behavior therapy had no sustainable efficacy (Lee et al., 2012). Overall, pharmacotherapy seemed more effective than psychosocial intervention (King et al., 2006).

Alternative/complementary treatments interventions of ADHD have not yet been accepted by the US Food and Drug Administration. However, some parents refuse regular treatments (Wilcox et al., 2007) and instead prefer alternative/complementary options (Karpouzis et al., 2010; Tzang et al., 2013). In this study, we regarded the pure cognition training (Cortese et al., 2015), hippotherapy (Oh et al., 2018), fluoxetine hydrochloride (Van Waes et al., 2012), cinnamon aromatherapy (Chen and Chen, 2008), EEG biofeedback (Chen et al., 2004), and sandplay therapy (Qiaomin et al., 2010) as alternative/complementary treatments of ADHD.

A recent network meta-analysis showed that psychostimulant treatment was more effective than placebo by indirectly estimating the relative effects or a single pooled treatment effect estimate of various interventions (Catala-Lopez et al., 2017). Instead of indirect estimation, interaction analysis can show valid inferences and direct statistical contrasts between groups. Therefore, a better updated meta-regression was recommended to directly compare the effects of ADHD treatments by systemically reviewing the “interaction analysis” of pharmacotherapy (psychostimulant and non-stimulant pharmacotherapy), psychosocial intervention, and alternative/complementary approach.

Meta-regression is an effective tool for exploratory analyses of heterogeneity and for studies of cross-level interactions (Bangdiwala et al., 2016). The heterogeneity can be reduced through interaction analysis (stratified or sub-group analysis) (Wang and Ware, 2013). Meta-regression can merge meta-analysis and linear regression principles to better clarify the linear relationship of various outcome measures and thereby provide clinicians and healthcare decision makers with more valuable information than meta-analysis (Baker et al., 2009). In addition, different scales to measure ES may lead to inconsistent results (Furukawa et al., 2005; Catala-Lopez et al., 2017). Unlike the studies on medication treatment, where the ratings by parents and teacher were usually adopted as the outcome measures, psychosocial treatment studies frequently utilized a broader array of outcome measures (e.g., parent and teacher ratings, observations of child behavior and parenting behavior, academic outcome). Due to some limitations of the ordinary meta-analysis, “updated meta-analysis” has been expected to synthesize comparative outcomes for different comparisons between psychosocial and pharmacological/combined approaches (Fabiano et al., 2015). Meta-regression is a statistical method that can be implemented following a traditional meta-analysis and regarded as its extension (Kelley and Kelley, 2012). Furthermore, meta-regression is currently the only technique to overcome invalid comparisons by merging meta-analysis of randomized controlled trials and the use of combined data (linear regression principle) to increase the statistical power of analysis from heterogeneity sources (Baker et al., 2009).

Importantly, meta-regression of randomized controlled trials can provide clinicians with the confidence to choose the right treatment option for ADHD (Impellizzeri and Bizzini, 2012; Rubinstein et al., 2019). This meta-regression aims to determine ES of stimulant pharmacotherapy, non-stimulant pharmacotherapy, parental behavior therapy (PBT), combination therapy, and alternative/complementary interventions. According to “partial regression plots” in the linear regression course of applied statistics, this meta-regression aimed to determine the ES of stimulant pharmacotherapy (MPH and lisdexamfetamine), non-stimulant pharmacotherapy (ATX and alpha-2 agonists), parental behavior therapy(PBT), combination therapy(psychostimulant plus PBT), and alternative/complementary interventions, after adjusting confounding factors of ADHD treatment type, study type, age, type of symptom scale, and publication year.

## Methods

### Eligibility Criteria

Inclusion criteria included the following: a formal diagnosis of ADHD, attention-deficit disorder, or hyperkinetic disorder with any subtype being diagnosed in accordance with either DSM-IV or ICD-10 criteria. Exclusion criteria included the following: patients with ADHD and a major neurological impairment, psychosis, major depressive disorder, or history of substance abuse disorder.

### Information Sources

Two authors (Y.C.C. and R.F.T.) independently searched the MEDLINE and PubMed databases (National Center for Biotechnology Information) from January 1980 to July 2018 for studies evaluating the efficacy and clinical outcomes of pharmacotherapeutic and non-pharmacotherapeutic interventions for children and adolescents with ADHD.

### Search and Study Selection

The following keywords were used to identify relevant articles: (clonidine OR guanfacine OR alpha 2 agonist* OR methylphenidate OR dextroamphetamine OR atomoxetine) AND (attention deficit OR attention-deficit OR “attention-deficit disorder with hyperactivity” OR ADHD OR ADD OR inattentive OR “hyperactive*” OR hyperkinetic OR impulsivity*) AND (“treat*” OR “intervention*” OR “therapy*” OR “psychotherapy*” OR “training*” OR “program*” OR “workshop*”).

Papers that satisfied the inclusion criteria were selected for comparing the treatment effects of pharmacotherapy, PBT, combined intervention, and complementary and alternative therapies for ADHD.

### Data Collection Process

The following information was extracted from each study: the last name of the first author, publication year, treatment type (pharmacotherapy, behavior therapy, combined intervention, or complementary and alternative therapies), primary outcome measurement, baseline and endpoint mean and SD of primary efficacy measures, mean age of total number of participants in the study, type of symptom measurement scale, and study quality.

The authors were contacted if data were missing, incomplete, or unclear. Only the available data were analyzed, without imputing the missing data. Studies with insufficient data were excluded (Table 1).

**Table 1.** Characteristic of Included Papers

Study	Year	Clinical studies included, No.	Mean age, y	Participants, No.	JADAD score
Cahill	2014	21	7.70 ~ 17.33	1126	—
Charach	2013	14	3.00 ~ 5.33	691	—
Maia	2014	7	8.20 ~ 9.84	348	—
Kelsey	2004	1	9.47	186	4
Huang	2015	1	8.40	97	NR
Bangs	2008	1	9.56	221	3
Michelson	2001	1	11.19	292	5
Handen	2015	1	8.13	99	5
Reichow	2013	7	4.80 ~ 10.00	222	2 ~ 5
Hirota	2014	11	9.20 ~ 12.60	2137	—
Cortese	2015	13	6.63 ~ 14.50	677	—
Tang	2007	6	9.50 ~ 10.50	1217	3 ~ 4
Ghuman	2009	1	5.02	12	NR
Biederman	2007	3	9.91	345	2
Newcorn	2006	1	10.55	224	2
Fan	2011	1	10.00	66	NR
Gu	2013	1	8.50	34	NR
Mohammadi	2016	1	9.00	47	3
MTA	1999	1	8.50	579	5
Golubchik	2018	1	10.09	28	1
Winters	2018	1	12.00	22	NR
Yunhye	2018	1	8.16	32	1
Ghajar	2018	1	8.28	25	5
Gamli	2018	1	14.90	82	NR
Newcorn	2017	1	14.70	807	3
Chen	2007	1	10.01	33	2
Lin	2007	1	8.63	76	1
Chen	2008	1	4.02	20	1
Jiang	2008	1	10.40	20	NR
Zhang	2009	1	10.55	20	NR
Cao	2009	1	10.80	28	NR
Wang	2010	1	9.00	30	2
Rejani	2012	1	7.50	40	1

Abbreviations: NR, non-randomized study.

### Data Items: Type of ADHD Treatment Options

We included and re-coded the following interventions: (1) Treat_1, pharmacotherapy with stimulant: MPH, lisdexamfetamine; (2) Treat_2, pharmacotherapy with non-stimulant: ATX, alpha-2 agonist (clonidine or guanfacine); (3) Treat_3, PBT; (4) Treat_4, combined intervention: psychostimulant + PBT; and (5) Treat_5, other (complementary or alternative therapies): cognition training, hippotherapy, fluoxetine hydrochloride, cinnamon aromatherapy, EEG biofeedback, and sandplay therapy.

The standardized mean difference (SMD) is scaleless in statistics. The current meta-regression therefore created a model to describe the linear relationship between (both continuous and categorical) study-level covariates and the ES. We applied the particular definition of SMD used in Cochrane reviews for the ES known in social science as Hedges’ (adjusted) *g* to express the size of the intervention effect in each study relative to the variability observed in that study.

### Data Items: Type of Symptom Measurement Scale

ES can be also influenced by the measurements used. To explore potentially influential factors and reduce heterogeneity, the primary scales used to evaluate the ES were classified and coded as follows: (1) Scale_1: Swanson, Nolan, and Pelham–IV, IOWA Conners Rating Scale hyperactivity; (2) Scale_2: Conners Parent (or Teacher) Rating Scale; (3) Scale_3: ADHD Rating Scale-IV, ADHD Rating Scale, Daily Parent Rating of Evening and Morning Behavior Scale; (4) Scale_4: Disruptive Behavior Disorder Rating Scale, Conduct Disorder Score, Irritability Scale, Turgay DSM-IV-Based Child and Adolescent Behavior Disorders Screening and Rating Scale, Child Behavior Check List Chinese, Aberrant Behavior Checklist, Barkley Home Situations Questionnaire, Barkley School Situations Questionnaire, Child Behavior Checklist, Difficulties in Emotion Regulation Scale, Swanson, Nolan, and Pelham–IV Oppositional Defiant Disorder, Eyberg Child Behavior Inventory; (5) Scale_5: Other (e.g., reaction time, Home Situations Questionnaire, Conners Continuous Performance Test II, Wechsler Memory Scale, Integrated Visual and Auditory Continuous Performance Test, mid-year school report cards). SMD is used as a summary statistic in meta-analysis when the studies assess the same outcome in a variety of ways (e.g., all the studies measured ADHD but adopted different psychometric scales). As described previously, there were 5 types of coded scales for evaluating the treatment effects. In this circumstance, it was necessary to standardize their measurements to a uniform scale before their combination.

### Synthesis of Results: Types of Study

The SMD has been the most commonly used ES for evaluating treatment effect in randomized control trials. However, SMD is significantly influenced by the choice of the control group, for example, an active (e.g., MPH vs alpha-2 agonist) or placebo control. In the study designs that used 2 or more treatments and compared with either an active or placebo control group, the data were extracted by dividing the individual studies into several appropriate and comparable groups.

To enable comparison, we classified the types of studies as follows: (1) StudyType_1: for placebo control studies with both pre- and post-tests in both groups, the SMD of Hedges’ *g* was calculated using the change-related information (from pre-test to post-test) in both treatment and control groups. We treated active control studies as having 2 independent groups with pre- and post-test information available and calculated each group’s unbiased SMD of Hedges’ *g* according to StudyType_2. (2) StudyType_2: single group with pre- and post-test information available. We followed the recommendation by Rosenthal and used a conservative estimate of *r* = .70 (Rosenthal, 1984). We then used the mean changes and the standard errors of the changes to calculate the unbiased SMD of Hedges’ *g*. (3) StudyType_3: 2 groups with only post-test information available. The unbiased SMD of Hedges’ *g* was calculated in terms of SD of change of the means and SDs of post-test from both treatment and control groups. (4) StudyType_4: a meta-analysis paper with only an unbiased SMD of Hedges’ *g* and its standard errors. In this study, we treated the types of study as a potential confounding variable.

### Quality Assessment and Risk of Bias in Individual Studies

According to the Jadad scale guidelines (Oxford quality scoring system), the quality of the randomized controlled trials can be shown by Jadad scale. A score of 1 represents that it is easy to use, 2 means it contains many of the important elements that have been empirically shown to correlate with bias, and 3 indicates that it has known reliability and external validity (Stephen et al., 2005). Here, 2 authors (K.H.Y. and Y.C.C.) evaluated the quality of the studies. The included trials were sorted and scored according to randomization (0, 1, or 2), double blinding (0, 1, or 2), and recording of dropouts and/or withdrawals (0 or 1); a score ≥3 was indicative of high quality (Jadad et al., 1996). Non-randomized studies were coded as “NR” and excluded from evaluation of impact on treatment effect.

The risk of bias was evaluated by funnel plots and Egger’s regression intercept tests. The goodness-of-fit indices of the fitted model were presented by 2 values and 1 plot; I-squared residual (residual variation due to heterogeneity), adjusted R-squared (the proportion of between-study variance explained by the model), and meta-regression plot.

### Summary Measures

Meta-regression is an extension to subgroup analyses that allows the effect of continuous, as well as categorical, characteristics to be investigated and in principle allows the effects of multiple factors to be investigated simultaneously. Therefore, even the continuous, as well as categorical, characteristics were investigated to explore their unbiased efficacy.

The primary outcomes of all included studies contained various types of evaluation scales. As described previously, there were 5 types of coded scales used to evaluate treatment effects. Each scale type featured its unique format, but all had the same evaluation purpose for ADHD treatment. SMD is used as a summary statistic in meta-analysis when the studies all assess the same outcome but measure it in a variety of ways. ES obtained from different evaluation scales were transferred into the Hedges’ *g*, where a larger ES value represented more improvement. Such conversions aided interpretation of the meta-regression.

The independent variables were publication year, mean age, and the abovementioned re-coded treatment types (Treat_1 to Treat_5), scale types (Scale_1 to Scale_5), and study design (StudyType_1 to StudyType_4). The level of significance was set at *P* < .05.

### Statistical Analysis

All meta-analyses were conducted using STATA v.13.0 software (StataCorp, College Station, TX, USA). We first used the fixed effects meta-analysis to evaluate the pooled ES. Random effects meta-analysis followed if inter-study heterogeneity was highly significant.

Following the meta-analysis of random effects, the meta-regression analyses were used to explore and compare factors potentially influencing treatment efficacy. The dependent variable was the SMD of Hedges’ *g*, assessed according to the aforementioned 4 types of study design. The independent variables were publication year, mean age, and abovementioned re-coded treatment types (Treat_1 to Treat_5), scale types (Scale_1 to Scale_5), and type of study design (StudyType_1 to StudyType_4). The level of significance was set at *P* < .05.

## Results

**Figure 1.**
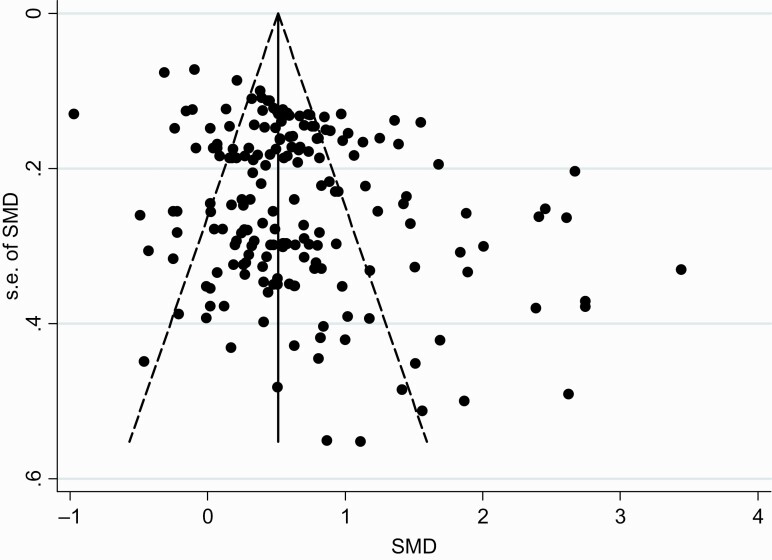
Funnel plot.

A total of 107 trials (reported in 33 papers, n = 9883 participants) met the inclusion criteria and had data amenable to analysis. The mean age ranged from 3 to 17.33 years. Owing to various treatment methodologies and/or treatment effect assessments included, the results were unsurprisingly heterogeneous. The heterogeneity of the meta-analysis was found to be highly significant (chi-squared = 1583.91, *d.f. = *186, *P* < .001). The index of variation in ES attributable to heterogeneity, I-squared, was 88.3%. To account for the heterogeneity among the studies, the random effect’s meta-analysis was used to estimate the pooled ES. The estimated pooled ES was equal to 0.642 with 95% confidence interval = [0.557, 0.726] and the estimate of between-study variance, tau-squared = 0.2826. The results of the funnel plot showed that there was a mild-moderate publication bias (Figure 1). The result of Egger’s test for small study effects was significant (*P* < .001).

We further employed the meta-regression analysis to investigate the possible sources of heterogeneity among the 107 included trials. The results of univariate meta-regression analysis are presented in Table 2 (with other factors’ effects being ignored): (1) the ES significantly increased gradually with respect to publication year (ES = 0.014 units/year, *P* = .031); (2) the ES decreased gradually with respect to the mean age of ADHD children, although it only reached borderline significance (ES = −0.041, *P* = .058); (3) the ES of Treat_2, Treat_3, and Treat_5 were, on average, 0.092, 0.030, and 0.151 units lower than that of Treat_1, respectively, although all of these results were not statistically significant. On the other hand, the ES of Treat_4 was 0.256 units higher than that of Treat_1, with *P* = .140 (insignificant).

**Table 2.** Results of Univariate Meta-Regression Analysis

SMD	Coefficients	Std. err.	*t*	*P* value	95% Confidence interval	
Treat_2 vs Treat_1	−0.092	0.124	−0.740	.457	−0.337	0.152
Treat_3 vs Treat_1	−0.030	0.153	−0.200	.842	−0.331	0.270
Treat_4 vs Treat_1	0.256	0.173	1.400	.140	−0.005	0.597
Treat_5 vs Treat_1	−0.151	0.150	−1.010	.314	−0.447	0.144
Scale_2 vs Scale_1	0.807	0.197	4.10	<.001	0.418	1.195
Scale_3 vs Scale_1	0.028	0.123	0.230	.814	−0.213	0.271
Scale_4 vs Scale_1	0.010	0.141	0.070	.942	−0.270	0.290
Scale_5 vs Scale_1	−0.434	0.139	−3.120	.002	−0.709	−0.156
StudyType_2 vs StudyType_1	0.435	0.108	4.020	<.001	0.221	0.649
StudyType_3 vs StudyType_1	0.107	0.140	0.770	.444	−0.160	0.383
StudyType_4 vs StudyType_1	−0.083	0.147	−0.560	.573	−0.373	0.208
Publication Year	0.015	0.007	2.280	.024	0.002	0.029
Age	−0.042	0.021	−1.950	.053	−0.084	0.001

Abbreviations: SMD: Standardized Mean Difference; Std. Err.: Standard Error; Scale_1:Swanson, Nolan, and Pelham–IV (SNAP-IV); Scale_2: Conners’ Parent (or Teacher) Rating Scale (CPRS or CTRS); Scale_3: ADHD Rating Scale-IV (ADHD-RS), ARS (ADHD Rating Scale), DPREMB-R (the Daily Parent Rating of Evening and Morning Behavior Scale); Scale_4: Disruptive Behavior Disorder rating scale (DBD-RS), conduct disorder score (20), Irritability scale, *T-DSM-IV-S* (Turgay *DSM-IV*-Based Child and Adolescent Behavior Disorders Screening and Rating Scale), CBCL_C (the Child Behavior Check List_Chinese), ABC (Aberrant Behavior Checklist), HSQ (Barkley’s Home Situations Questionnaire), SSQ (Barkley’s School Situations Questionnaire), CBCL (Child Behavior Checklist), DERS (Difficulties in Emotion Regulation Scale), SNAP-IV ODD, ECBI (Eyberg Child Behaviour Inventory); Scale_5: Others: (Reaction time, HSQ (Home Situations Questionnaire), CPT-II (Conners’ Continuous Performance Test II), WMS (Wechsler Memory Scale ), IVA-CPT (Integrated Visual and Auditory- Continuous Performance Test); Mid-year school report cards.Treat_1: METHYLPHENIDATE (MPH) or Lisdexamfetamine; Treat_2: Atomoxetine (ATX) or Alpha-2 agonist (clonidine) or Guanfacine; Treat_3: Parents Behavior Training (PBT); Treat_4: Medication (MPH, ATX or Alpha-2) + PTB (parents behavior training); Treat_5: Others (Cognition Training, Hippotherapy, Fluoxetine Hydrochloride, Cinnamon aromatherapy, EEG Biofeedback, Sandplay Therapy); *t: t* distribution.

The impacts of types of treatment, types of evaluation scale, and types of study on treatments efficacy were mutually affected (*P* values of all 3 Fisher’s exact tests < .001, not shown). Therefore, to evaluate the impact of any 1 of these 3 factors on treatment efficacy, we simultaneously adjusted for the effects of other 2 factors.

Accordingly, to compare the treatment effects among all collected intervention methods after adjusting for the effects of other potential factors (types of scale, types of study, publication years, and mean age), the multiple meta-regression analysis was used (Table 3). After adjusting for the effects of publication year, age, types of evaluation scale, and types of study, the treatment effect (in terms of ES) of Treat_1 (MPH or lisdexamfetamine) was the highest among the 5 classified treatments. More specifically, compared with Treat_1, the ES of Treat_2 (ATX or Alpha-2 agonist [clonidine] or guanfacine) and Treat_5 (Others) were 0.384 and 0.419 units, respectively, significantly less than that of Treat_1 (*P*  = .004 and .028, respectively). It is worth mentioning that the PBT alone (Treat_3, PBT) and combined treatment (Treat-4, Medication + PBT) were 0.308 and 0.196 units less effective, respectively, than Treat_1, although the results were insignificant (*P*  = .095 and .209, respectively).

**Table 3.** Results of Multiple Meta-Regression Analysis

SMD	Coefficients	Std. Err.	*t*	*P* value	95% Confidence Interval	
Treat_2 vs Treat_1	−0.384	0.133	−2.880	.004	−0.648	−0.121
Treat_3 vs Treat_1	−0.308	0.183	−1.600	.095	−0.670	0.054
Treat_4 vs Treat_1	−0.196	0.156	−1.260	.209	−0.504	0.111
Treat_5 vs Treat_1	−0.419	0.190	−2.210	.028	−0.794	−0.045
Scale_2 vs Scale_1	0.750	0.190	3.950	<.001	0.375	1.124
Scale_3 vs Scale_1	0.384	0.139	2.750	.007	0.109	0.659
Scale_4 vs Scale_1	−0.085	0.143	−0.600	.552	−0.368	0.198
Scale_5 vs Scale_1	−0.504	0.150	−3.360	.001	−0.801	−0.208
StudyType_2 vs StudyType_1	0.333	0.113	2.940	.004	0.110	0.556
StudyType_3 vs StudyType_1	0.408	0.149	0.270	.785	−0.253	0.335
StudyType_4 vs StudyType_1	−0.199	0.188	−1.060	.291	−0.570	0.172
Publication Year	0.003	0.007	0.480	.631	−0.011	0.017
Age	−0.059	0.025	−2.330	.021	−0.109	−0.009

Abbreviations: SMD: Standardized Mean Difference; Std. Err.: Standard Error; Scale_1:Swanson, Nolan, and Pelham–IV (SNAP-IV); Scale_2: Conners’ Parent (or Teacher) Rating Scale (CPRS or CTRS); Scale_3: ADHD Rating Scale-IV (ADHD-RS), ARS (ADHD Rating Scale), DPREMB-R (the Daily Parent Rating of Evening and Morning Behavior Scale); Scale_4: Disruptive Behavior Disorder rating scale (DBD-RS), conduct disorder score (20), Irritability scale, *T-DSM-IV-S* (Turgay *DSM-IV*-Based Child and Adolescent Behavior Disorders Screening and Rating Scale), CBCL_C (the Child Behavior Check List_Chinese), ABC (Aberrant Behavior Checklist), HSQ (Barkley’s Home Situations Questionnaire), SSQ (Barkley’s School Situations Questionnaire), CBCL (Child Behavior Checklist), DERS (Difficulties in Emotion Regulation Scale), SNAP-IV ODD, ECBI (Eyberg Child Behaviour Inventory); Scale_5: Others: (Reaction time, HSQ (Home Situations Questionnaire), CPT-II (Conners’ Continuous Performance Test II), WMS (Wechsler Memory Scale ), IVA-CPT (Integrated Visual and Auditory- Continuous Performance Test); Mid-year school report cards.Treat_1: METHYLPHENIDATE (MPH) or Lisdexamfetamine; Treat_2: Atomoxetine (ATX) or Alpha-2 agonist (clonidine) or Guanfacine; Treat_3: Parents Behavior Training (PBT); Treat_4: Medication (MPH, ATX or Alpha-2) + PTB (parents behavior training); Treat_5: Others (Cognition Training, Hippotherapy, Fluoxetine Hydrochloride, Cinnamon aromatherapy, EEG Biofeedback, Sandplay Therapy); *t: t* distribution.

**Figure 2.**
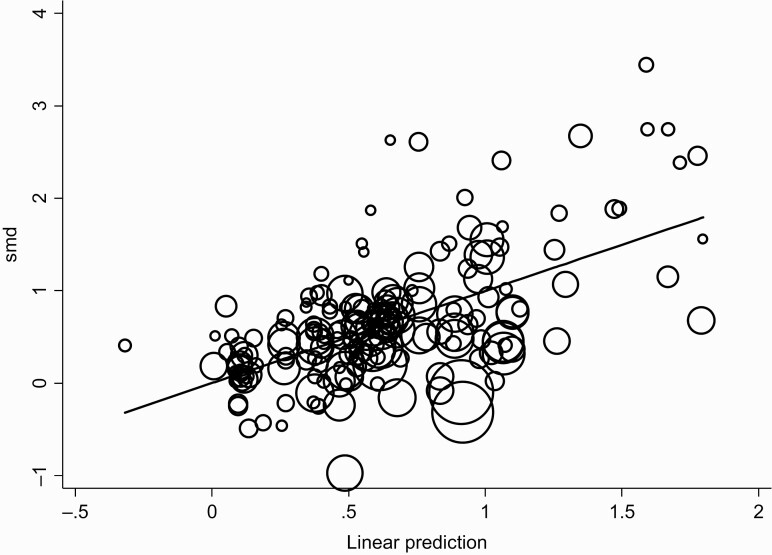
The meta-regression plot of SMD as a function of the linear predicted values (adjusted *R*^2^ = 35.22%); the circles are in proportion to the study weights in the meta-regression.

The corresponding residual variation due to the heterogeneity for this meta-regression, the I-squared residual, was 85.97%. The proportion of between-study variance explained by the meta-regression model, adjusted R-squared, was 35.22%. The corresponding meta-regression plot is shown in Figure 2. The risk of bias was evaluated by funnel plots and Egger’s regression intercept tests for bias. The result of Egger’s test showed that the publication bias was significant (*t* = 4.48, *P* < .001) (Figure 1). In other words, there existed some potential publication bias in this study.

## Discussion

To date, clinicians struggle to balance benefits and costs of various interventions of ADHD (Wolraich et al., 2011, 2019). This meta-regression, including 9883 participants in 107 trials published on Medline and PubMed between January 1999 and July 2018, compared the ES of various treatments for ADHD in children and adolescents. These treatments included pharmacotherapy (psychostimulant and non-psychostimulant), psychosocial intervention (PBT), combined intervention (psychostimulant plus PBT), and other alternative/complementary therapies.

Previous studies on ADHD treatments sometimes met difficulties, such as heterogeneity across outcome measures and tests (Jadad et al., 1999). Catala-Lopez et al. (Catala-Lopez et al., 2017) analyzed 190 randomized controlled trials on ADHD among children and adolescents until 2016. They demonstrated that pharmacotherapy was more effective than a placebo and suggested that more updated meta-analysis would be needed for managing heterogeneity among studies. Therefore, the current meta-regression overcame the heterogenous nature of various treatments and focused on 107 randomized controlled trials, which were reported in 33 papers.

The findings of the current study appear clinically instructive. After adjusting for confounding variables, psychostimulant medication was significantly more effective than non-stimulant treatment (*P* = .004) and complementary and alternative intervention (*P* = .028) (Table 2). However, psychostimulant therapy did not significantly differ from PBT or combination therapy of psychostimulant and PBT (Table 2). Also, diagnosis and pharmacotherapy of ADHD may have varied with the publication years, which, therefore, impacted the ES. In accordance, publication year as well as race, means of statistical analysis, study design, publication year, performing year, and geographical setting were found to confound the effect estimate in a previous study (Blettner et al., 2014). Likewise, the publication year also influenced the ES of a depression study (Juliane and Peter, 2012). We also found that, ignoring other factors’ effects, the ES significantly increased with respect to the publication year (ES = 0.015 units/year, *P* = .024). Therefore, the current meta regression adjusted the confounder of publication year to obtain an unbiased ES.

In addition to psychostimulants (Currie et al., 2014; Visser et al., 2016), parent-based interventions have been effective in improving behaviors of children with externalizing behavior problems (Mingebach et al., 2018). Behavior psychotherapy protocols have shown their values in improving complicated emotional symptoms of children (Ptacek et al., 2014; Wang et al., 2014). For ADHD children with oppositional defiant disorder, disruptive mood dysregulation disorder, and stressful parent-child relationships, combining psychostimulants with PBT may be needed (Latimer et al., 2012). Past research also showed that PBT was able to enhance pharmacotherapy by increasing positive interaction and ensuring good healthcare quality (Mulqueen et al., 2015; Lange et al., 2016).

Since the remission rate from pharmacotherapy by osmotic-release oral system-MPH remains between 44% (Swanson and Hechtman, 2005) and 66.1% (Chou et al., 2009), whether combining pharmacotherapy with PBT or psychoeducation can increase the remission rate deserves study. Treatment compliance may be increased and parenting stress relieved if pharmacotherapy to change symptoms and parenting programs to change behavior and emotional disturbance are combined (Wang et al., 2014). Future randomized controlled studies are needed to compare the efficacy of different parental interventions for ADHD. However, unlike studies of pharmacotherapy for ADHD, where only parents’ and teachers’ ratings of ADHD symptoms were primarily used as outcome measures, studies of psychosocial treatments such as PBT utilized a broader array of outcome measures (e.g., parent and teacher ratings, observations of child behavior and parenting behavior, academic outcome). Therefore, it has been suggested that “updated meta-analysis” can be used to synthesize comparative outcomes across different measurements between psychosocial and pharmacological/combined approaches (Fabiano et al., 2015). The current study is valuable in overcoming the heterogeneity problem to explore the effect estimates of various treatment interventions. Such an updated meta regression study may provide ADHD clinician with more evidence in choosing suitable therapy modalities for ADHD.

### Limitations

There were some limitations in this study. The mean age of the study population ranged from 5.2 to 17.7 years. There may have been informant rater problems affecting the treatment effects (Sonuga-Barke et al., 2013). We planned to identify all treatment options across Western and Eastern practices, but articles in local languages may have been missed. Most of the alternative therapies included here were conducted in China; the generalizability of the findings remains uncertain. In addition, we regarded stimulants and non-stimulants as pharmacotherapy for ADHD. Fluoxetine is not allowed for treatment of ADHD, while some studies used it as an alternative intervention for ADHD in children. We also regarded it as one of the alternative treatments. Partly because only methylphenidate has been available in Taiwan, in the current study, stimulant treatments were represented by methylphenidate and lisdexamfetamine instead of other stimulant medication for ADHD commonly used in The States like Dextroamphetamine, Adderall. Finally, we did not analyze differences between short- and long-acting formulations or dosage and intensity of pharmacological and non-pharmacological treatments.

## Conclusions

Although there are many therapies available for ADHD, comparative efficacy for different treatments remains unclear based on previous meta-analyses. The current study systematically meta-regressed the ES of various treatments for ADHD and overcame heterogeneity among ADHD studies. It is also the first, to our knowledge, to attempt to examine all published meta-analyses and randomized control trials on multitudinous treatments for ADHD. The results showed that the psychostimulant surpassed non-stimulant pharmacotherapy and alternative/complementary interventions. Psychostimulant therapy, PBT, and a combination of psychostimulant and PBT appeared to be similar in their efficacy according to this meta-regression. Our findings will help clinicians, healthcare providers, parents, and caregivers in choosing treatment for ADHD in children or adolescents.

## Supplementary Material

pyab034_suppl_Supplementary_Matrials

## References

[CIT0001] Asherson P , Bushe C, Saylor K, Tanaka Y, Deberdt W, Upadhyaya H (2014) Efficacy of atomoxetine in adults with attention deficit hyperactivity disorder: an integrated analysis of the complete database of multicenter placebo-controlled trials. J Psychopharmacol 28:837–846. 25035246 10.1177/0269881114542453 PMC4230847

[CIT0002] Asherson P , Stes S, Nilsson Markhed M, Berggren L, Svanborg P, Kutzelnigg A, Deberdt W (2015) The effects of atomoxetine on emotional control in adults with ADHD: an integrated analysis of multicenter studies. Eur Psychiatry 30:511–520. 25649490 10.1016/j.eurpsy.2014.12.002

[CIT0003] Atkinson M , Hollis C (2010) NICE guideline: attention deficit hyperactivity disorder. Arch Dis Child Educ Pract Ed 95:24–27. 20145015 10.1136/adc.2009.175943

[CIT0004] Baker WL , White CM, Cappelleri JC, Kluger J, Coleman CI; Health Outcomes, Policy, and Economics (HOPE) Collaborative Group (2009) Understanding heterogeneity in meta-analysis: the role of meta-regression. Int J Clin Pract 63:1426–1434. 19769699 10.1111/j.1742-1241.2009.02168.x

[CIT0005] Banaschewski T , Coghill D, Santosh P, Zuddas A, Asherson P, Buitelaar J, Danckaerts M, Dopfner M, Faraone SV, Rothenberger A, Sergeant J, Steinhausen HC, Sonuga-Barke EJ, Taylor E (2008) [Long-acting medications for the treatment of hyperkinetic disorders - a systematic review and European treatment guidelines. Part 2: a quantitative evaluation of long-acting medications]. Z Kinder Jugendpsychiatr Psychother 36:97–106; quiz 106–107. 18622939 10.1024/1422-4917.36.2.97

[CIT0006] Bangdiwala SI , Bhargava A, O’Connor DP, Robinson TN, Michie S, Murray DM, Stevens J, Belle SH, Templin TN, Pratt CA (2016) Statistical methodologies to pool across multiple intervention studies. Transl Behav Med 6:228–235. 27356993 10.1007/s13142-016-0386-8 PMC4927450

[CIT0007] Bangs ME , Hazell P, Danckaerts M, Hoare P, Coghill DR, Wehmeier PM, Williams DW, Moore RJ, Levine L; Atomoxetine ADHD/ODD Study Group (2008) Atomoxetine for the treatment of attention-deficit/hyperactivity disorder and oppositional defiant disorder. Pediatrics 121:e314–e320. 18245404 10.1542/peds.2006-1880

[CIT0008] Biederman J , Petty CR, Dolan C, Hughes S, Mick E, Monuteaux MC, Faraone SV (2008) The long-term longitudinal course of oppositional defiant disorder and conduct disorder in ADHD boys: findings from a controlled 10-year prospective longitudinal follow-up study. Psychol Med 38:1027–1036. 18205967 10.1017/S0033291707002668

[CIT0009] Blettner M , Krahn U, Schlattmann P (2014) Handbook of epidemiology. 2nd ed. New York: Springer.

[CIT0010] Bloch MH (2014) Meta-analysis and moderator analysis: can the field develop further? J Am Acad Child Adolesc Psychiatry 53:135–137. 24472248 10.1016/j.jaac.2013.12.001 PMC4471847

[CIT0011] Briars L , Todd T (2016) A review of pharmacological management of attention-deficit/hyperactivity disorder. J Pediatr Pharmacol Ther 21:192–206. 27453697 10.5863/1551-6776-21.3.192 PMC4956327

[CIT0012] Brown RT , Amler RW, Freeman WS, Perrin JM, Stein MT, Feldman HM, Pierce K, Wolraich ML; American Academy of Pediatrics Committee on Quality Improvement; American Academy of Pediatrics Subcommittee on Attention-Deficit/Hyperactivity Disorder (2005) Treatment of attention-deficit/hyperactivity disorder: overview of the evidence. Pediatrics 115:e749–e757. 15930203 10.1542/peds.2004-2560

[CIT0013] Castells X , Ramon M, Cunill R, Olive C, Serrano D (2020) Relationship between treatment duration and efficacy of pharmacological treatment for ADHD: a meta-analysis and meta-regression of 87 randomized controlled clinical trials. J Atten Disord. doi: 1087054720903372. Online ahead of print. 10.1177/1087054720903372 32075485

[CIT0014] Catala-Lopez F , Hutton B, Núñez-Beltrán A, Page MJ, Ridao M, Macías Saint-Gerons D, Catalá MA, Tabarés-Seisdedos R, Moher D (2017) The pharmacological and non-pharmacological treatment of attention deficit hyperactivity disorder in children and adolescents: a systematic review with network meta-analyses of randomised trials. PLoS One 12:e0180355. 28700715 10.1371/journal.pone.0180355 PMC5507500

[CIT0015] Charach A , Carson P, Fox S, Ali MU, Beckett J, Lim CG (2013) Interventions for preschool children at high risk for ADHD: a comparative effectiveness review. Pediatrics 131:e1584–e1604. 23545375 10.1542/peds.2012-0974

[CIT0016] Chen HM , Chen HW (2008) The effect of applying cinnamon aromatherapy for children with attention deficit hyperactivity disorder. J Chin Med 19:27–34.

[CIT0017] Chen YX , Jiao GK, Wang CY, Ke XY, Wang MJ, Chen Y (2004) Therapeutic effectiveness of electroencephalography biofeedback on children with attention deficit hyperactivity disorder. Chin J Clin Rehabil 8:3690–3691.

[CIT0018] Chou WJ , Chou MC, Tzang RF, Hsu YC, Gau SS, Chen SJ, Wu YY, Huang YF, Liang HY, Cheng H (2009) Better efficacy for the osmotic release oral system methylphenidate among poor adherents to immediate-release methylphenidate in the three ADHD subtypes. Psychiatry Clin Neurosci 63:167–175. 19335386 10.1111/j.1440-1819.2009.01937.x

[CIT0019] Cinnamon Bidwell L , Dew RE, Kollins SH (2010) Alpha-2 adrenergic receptors and attention-deficit/hyperactivity disorder. Curr Psychiatry Rep 12:366–373. 20652773 10.1007/s11920-010-0136-4 PMC3676929

[CIT0020] Coghill DR , Seth S, Pedroso S, Usala T, Currie J, Gagliano A (2014) Effects of methylphenidate on cognitive functions in children and adolescents with attention-deficit/hyperactivity disorder: evidence from a systematic review and a meta-analysis. Biol Psychiatry 76:603–615. 24231201 10.1016/j.biopsych.2013.10.005

[CIT0021] Cortese S , Ferrin M, Brandeis D, Buitelaar J, Daley D, Dittmann RW, Holtmann M, Santosh P, Stevenson J, Stringaris A, Zuddas A, Sonuga-Barke EJ; European ADHD Guidelines Group (EAGG) (2015) Cognitive training for attention-deficit/hyperactivity disorder: meta-analysis of clinical and neuropsychological outcomes from randomized controlled trials. J Am Acad Child Adolesc Psychiatry 54:164–174. 25721181 10.1016/j.jaac.2014.12.010 PMC4382075

[CIT0022] Cunill R , Castells X, Tobias A, Capellà D (2013) Atomoxetine for attention deficit hyperactivity disorder in the adulthood: a meta-analysis and meta-regression. Pharmacoepidemiol Drug Saf 22:961–969. 23813665 10.1002/pds.3473

[CIT0023] Currie J , Stabile M, Jones L (2014) Do stimulant medications improve educational and behavioral outcomes for children with ADHD? J Health Econ 37:58–69. 24954077 10.1016/j.jhealeco.2014.05.002 PMC4815037

[CIT0024] De Crescenzo F , Cortese S, Adamo N, Janiri L (2017) Pharmacological and non-pharmacological treatment of adults with ADHD: a meta-review. Evid Based Ment Health 20:4–11. 27993933 10.1136/eb-2016-102415 PMC10699262

[CIT0025] Dittmann RW , Schacht A, Helsberg K, Schneider-Fresenius C, Lehmann M, Lehmkuhl G, Wehmeier PM (2011) Atomoxetine versus placebo in children and adolescents with attention-deficit/hyperactivity disorder and comorbid oppositional defiant disorder: a double-blind, randomized, multicenter trial in Germany. J Child Adolesc Psychopharmacol 21:97–110. 21488751 10.1089/cap.2009.0111

[CIT0026] Fabiano GA , Schatz NK, Aloe AM, Chacko A, Chronis-Tuscano A (2015) A systematic review of meta-analyses of psychosocial treatment for attention-deficit/hyperactivity disorder. Clin Child Fam Psychol Rev 18:77–97. 25691358 10.1007/s10567-015-0178-6 PMC4346344

[CIT0027] Faraone SV , Buitelaar J (2010) Comparing the efficacy of stimulants for ADHD in children and adolescents using meta-analysis. Eur Child Adolesc Psychiatry 19:353–364. 19763664 10.1007/s00787-009-0054-3

[CIT0028] Furukawa TA , Cipriani A, Barbui C, Brambilla P, Watanabe N (2005) Imputing response rates from means and standard deviations in meta-analyses. Int Clin Psychopharmacol 20:49–52. 15602117 10.1097/00004850-200501000-00010

[CIT0029] Golubchik P , Hamerman H, Manor I, Peskin M, Weizman A (2018) Effectiveness of parental training, methylphenidate treatment, and their combination on academic achievements and behavior at school of children with attention-deficit hyperactivity disorder. Int Clin Psychopharmacol 33:229–232. 29608460 10.1097/YIC.0000000000000218

[CIT0030] Group MC (1999) A 14-month randomized clinical trial of treatment strategies for attention-deficit/hyperactivity disorder. The MTA Cooperative Group. Multimodal Treatment Study of Children with ADHD. Arch Gen Psychiatry 56:1073–1086. 10591283 10.1001/archpsyc.56.12.1073

[CIT0031] Hall CL , Taylor JA, Newell K, Baldwin L, Sayal K, Hollis C (2016) The challenges of implementing ADHD clinical guidelines and research best evidence in routine clinical care settings: Delphi survey and mixed-methods study. Bjpsych Open 2:25–31. 27703750 10.1192/bjpo.bp.115.002386 PMC4995556

[CIT0032] Handen BL , Aman MG, Arnold LE, Hyman SL, Tumuluru RV, Lecavalier L, Corbett-Dick P, Pan X, Hollway JA, Buchan-Page KA, Silverman LB, Brown NV, Rice RR Jr, Hellings J, Mruzek DW, McAuliffe-Bellin S, Hurt EA, Ryan MM, Levato L, Smith T (2015) Atomoxetine, parent training, and their combination in children with autism spectrum disorder and attention-deficit/hyperactivity disorder. J Am Acad Child Adolesc Psychiatry 54:905–915. 26506581 10.1016/j.jaac.2015.08.013 PMC4625086

[CIT0033] Hirota T , Schwartz S, Correll CU (2014) Alpha-2 agonists for attention-deficit/hyperactivity disorder in youth: a systematic review and meta-analysis of monotherapy and add-on trials to stimulant therapy. J Am Acad Child Adolesc Psychiatry 53:153–173. 24472251 10.1016/j.jaac.2013.11.009

[CIT0034] Huang YH , Chung CY, Ou HY, Tzang RF, Huang KY, Liu HC, Sun FJ, Chen SC, Pan YJ, Liu SI (2015) Treatment effects of combining social skill training and parent training in Taiwanese children with attention deficit hyperactivity disorder. J Formos Med Assoc 114:260–267. 25777975 10.1016/j.jfma.2012.10.019

[CIT0035] Impellizzeri FM , Bizzini M (2012) Systematic review and meta-analysis: a primer. Int J Sports Phys Ther 7:493–503. 23091781 PMC3474302

[CIT0036] Jadad AR , Moore RA, Carroll D, Jenkinson C, Reynolds DJ, Gavaghan DJ, McQuay HJ (1996) Assessing the quality of reports of randomized clinical trials: is blinding necessary? Control Clin Trials 17:1–12. 8721797 10.1016/0197-2456(95)00134-4

[CIT0037] Jadad AR , Boyle M, Cunningham C, Kim M, Schachar R (1999) Treatment of attention-deficit/hyperactivity disorder. Evid Rep Technol Assessi–viii:1–341. PMC4782276 10790990

[CIT0038] Juliane E , Peter S (2012) The effects of mindfulness meditation: a meta-analysis. Mindfulness 3:174–189.

[CIT0039] Kambeitz J , Romanos M, Ettinger U (2014) Meta-analysis of the association between dopamine transporter genotype and response to methylphenidate treatment in ADHD. Pharmacogenomics J 14:77–84. 23588108 10.1038/tpj.2013.9

[CIT0040] Karpouzis F , Bonello R, Pollard H (2010) Chiropractic care for paediatric and adolescent attention-deficit/hyperactivity disorder: a systematic review. Chiropr Osteopat 18:13. 20525195 10.1186/1746-1340-18-13 PMC2891800

[CIT0041] Kelley GA , Kelley KS (2012) Statistical models for meta-analysis: a brief tutorial. World J Methodol 2:27–32. 25237614 10.5662/wjm.v2.i4.27 PMC4145560

[CIT0042] Kelsey DK , Sumner CR, Casat CD, Coury DL, Quintana H, Saylor KE, Sutton VK, Gonzales J, Malcolm SK, Schuh KJ, Allen AJ (2004) Once-daily atomoxetine treatment for children with attention-deficit/hyperactivity disorder, including an assessment of evening and morning behavior: a double-blind, placebo-controlled trial. Pediatrics 114:e1–e8. 15231966 10.1542/peds.114.1.e1

[CIT0043] King S , Griffin S, Hodges Z, Weatherly H, Asseburg C, Richardson G, Golder S, Taylor E, Drummond M, Riemsma R (2006) A systematic review and economic model of the effectiveness and cost-effectiveness of methylphenidate, dexamfetamine and atomoxetine for the treatment of attention deficit hyperactivity disorder in children and adolescents. Health Technol Assess 10:iii–iv, xiii. 10.3310/hta10230 16796929

[CIT0044] Knouse LE , Zvorsky I, Safren SA (2013) Depression in adults with attention-deficit/hyperactivity disorder (ADHD): the mediating role of cognitive-behavioral factors. Cognit Ther Res 37:1220–1232. 10.1007/s10608-013-9569-5 PMC4469239 26089578

[CIT0045] Lange AM , Daley D, Frydenberg M, Rask CU, Sonuga-Barke E, Thomsen PH (2016) The effectiveness of parent training as a treatment for preschool attention-deficit/hyperactivity disorder: study protocol for a randomized controlled, multicenter trial of the New Forest Parenting Program in everyday clinical practice. JMIR Res Protoc 5:e51. 27076496 10.2196/resprot.5319 PMC4848388

[CIT0046] Latimer K , Wilson P, Kemp J, Thompson L, Sim F, Gillberg C, Puckering C, Minnis H (2012) Disruptive behaviour disorders: a systematic review of environmental antenatal and early years risk factors. Child Care Health Dev 38:611–628. 22372737 10.1111/j.1365-2214.2012.01366.x

[CIT0047] Lee PC , Niew WI, Yang HJ, Chen VC, Lin KC (2012) A meta-analysis of behavioral parent training for children with attention deficit hyperactivity disorder. Res Dev Disabil 33:2040–2049. 22750360 10.1016/j.ridd.2012.05.011

[CIT0048] Maia CR , Cortese S, Caye A, Deakin TK, Polanczyk GV, Polanczyk CA, Rohde LA (2017) Long-term efficacy of methylphenidate immediate-release for the treatment of childhood ADHD. J Atten Disord 21:3–13. 25501355 10.1177/1087054714559643

[CIT0049] Michelson D , Faries D, Wernicke J, Kelsey D, Kendrick K, Sallee FR, Spencer T; Atomoxetine ADHD Study Group (2001) Atomoxetine in the treatment of children and adolescents with attention-deficit/hyperactivity disorder: a randomized, placebo-controlled, dose-response study. Pediatrics 108:E83. 11694667 10.1542/peds.108.5.e83

[CIT0050] Mingebach T , Kamp-Becker I, Christiansen H, Weber L (2018) Meta-meta-analysis on the effectiveness of parent-based interventions for the treatment of child externalizing behavior problems. PLoS One 13:e0202855. 30256794 10.1371/journal.pone.0202855 PMC6157840

[CIT0051] Mohammadi MR , Soleimani AA, Ahmadi N, Davoodi E (2016) A comparison of effectiveness of parent behavioral management training and methylphenidate on reduction of symptoms of attention deficit hyperactivity disorder. Acta Med Iran 54:503–509. 27701720

[CIT0052] Mulqueen JM , Bartley CA, Bloch MH (2015) Meta-analysis: parental interventions for preschool ADHD. J Atten Disord 19:118–124. 24071773 10.1177/1087054713504135

[CIT0053] Nutt DJ , Fone K, Asherson P, Bramble D, Hill P, Matthews K, Morris KA, Santosh P, Sonuga-Barke E, Taylor E, Weiss M, Young S; British Association for Psychopharmacology (2007) Evidence-based guidelines for management of attention-deficit/hyperactivity disorder in adolescents in transition to adult services and in adults: recommendations from the British Association for Psychopharmacology. J Psychopharmacol 21:10–41. 17092962 10.1177/0269881106073219

[CIT0054] Oh Y , Joung YS, Jang B, Yoo JH, Song J, Kim J, Kim K, Kim S, Lee J, Shin HY, Kwon JY, Kim YH, Jeong B (2018) Efficacy of hippotherapy versus pharmacotherapy in attention-deficit/hyperactivity disorder: a randomized clinical trial. J Altern Complement Med 24:463–471. 29641212 10.1089/acm.2017.0358

[CIT0055] Padilha SCOS , Virtuoso S, Tonin FS, Borba HHL, Pontarolo R (2018) Efficacy and safety of drugs for attention deficit hyperactivity disorder in children and adolescents: a network meta-analysis. Eur Child Adolesc Psychiatry 27:1335–1345. 29460165 10.1007/s00787-018-1125-0

[CIT0056] Polanczyk G , de Lima MS, Horta BL, Biederman J, Rohde LA (2007) The worldwide prevalence of ADHD: a systematic review and metaregression analysis. Am J Psychiatry 164:942–948. 17541055 10.1176/ajp.2007.164.6.942

[CIT0057] Ptacek R , Kuzelova H, Stefano GB, Raboch J, Kream RM, Goetz M (2014) ADHD and growth: questions still unanswered. Neuro Endocrinol Lett 35:1–6. 24625909

[CIT0058] Qiaomin W , Gang H, Xiaolei Z, Xiuling H, Dandan W (2010) Effects of sandplay therapy in children with attention deficit hyperactivity disorders. Chin Ment Health J 9:691–695.

[CIT0059] Reichow B , Volkmar FR, Bloch MH (2013) Systematic review and meta-analysis of pharmacological treatment of the symptoms of attention-deficit/hyperactivity disorder in children with pervasive developmental disorders. J Autism Dev Disord 43:2435–2441. 23468071 10.1007/s10803-013-1793-z PMC3787525

[CIT0060] Rosenthal R (1984) Defining Research Results, Meta-analytic procedures for social science research Beverly Hills. Beverly Hills, CA: Sage Publications.

[CIT0061] Rubinstein SM , de Zoete A, van Middelkoop M, Assendelft WJJ, de Boer MR, van Tulder MW (2019) Benefits and harms of spinal manipulative therapy for the treatment of chronic low back pain: systematic review and meta-analysis of randomised controlled trials. BMJ 364:l689. 30867144 10.1136/bmj.l689 PMC6396088

[CIT0062] Schwartz S , Correll CU (2014) Efficacy and safety of atomoxetine in children and adolescents with attention-deficit/hyperactivity disorder: results from a comprehensive meta-analysis and metaregression. J Am Acad Child Adolesc Psychiatry 53:174–187. 24472252 10.1016/j.jaac.2013.11.005

[CIT0063] Seyrek S , Cop E, Sinir H, Ugurlu M, Şenel S (2017) Factors associated with internet addiction: cross-sectional study of Turkish adolescents. Pediatr Int 59:218–222. 27507735 10.1111/ped.13117

[CIT0064] Sonuga-Barke EJ , et al.; European ADHD Guidelines Group. (2013) Nonpharmacological interventions for ADHD: systematic review and meta-analyses of randomized controlled trials of dietary and psychological treatments. Am J Psychiatry 170:275–289. 23360949 10.1176/appi.ajp.2012.12070991

[CIT0065] Stephen H , Halpern M, Joanne D (2005) Jadad scale for reporting randomized controlled trials. Oxford, UK: Blackwell Publishing Ltd.

[CIT0067] Swanson JM , Hechtman L (2005) Using long-acting stimulants: does it change ADHD treatment outcome? Can Child Adolesc Psychiatr Rev 14:2–3. PMC2547090 19030517

[CIT0068] Tzang RF , Chang YC, Chen CC (2013) Barriers to seeking help among children with attention deficit hyperactivity disorder in Taiwan. Asia Pac Psychiatry 6:373–378. 23857893 10.1111/appy.12064

[CIT0069] Van Waes V , Carr B, Beverley JA, Steiner H (2012) Fluoxetine potentiation of methylphenidate-induced neuropeptide expression in the striatum occurs selectively in direct pathway (striatonigral) neurons. J Neurochem 122:1054–1064. 22738672 10.1111/j.1471-4159.2012.07852.x PMC3423503

[CIT0070] Visser SN , Danielson ML, Wolraich ML, Fox MH, Grosse SD, Valle LA, Holbrook JR, Claussen AH, Peacock G (2016) Vital signs: national and state-specific patterns of attention deficit/hyperactivity disorder treatment among insured children aged 2-5 years - United States, 2008-2014. MMWR Morb Mortal Wkly Rep 65:443–450. 27149047 10.15585/mmwr.mm6517e1

[CIT0071] Wang CH , Mazursky-Horowitz H, Chronis-Tuscano A (2014) Delivering evidence-based treatments for child attention-deficit/hyperactivity disorder (ADHD) in the context of parental ADHD. Curr Psychiatry Rep 16:474. 25135774 10.1007/s11920-014-0474-8 PMC4664577

[CIT0072] Wang R , Ware JH (2013) Detecting moderator effects using subgroup analyses. Prev Sci 14:111–120. 21562742 10.1007/s11121-011-0221-x PMC3193873

[CIT0073] Wilcox CE , Washburn R, Patel V (2007) Seeking help for attention deficit hyperactivity disorder in developing countries: a study of parental explanatory models in Goa, India. Soc Sci Med 64:1600–1610. 17267087 10.1016/j.socscimed.2006.11.032

[CIT0074] Wolraich ML (2012) The new attention deficit hyperactivity disorder clinical practice guidelines published by the American Academy of Pediatrics. J Dev Behav Pediatr 33:76–77. 22218017 10.1097/DBP.0b013e318241eadf

[CIT0066] Wolraich M, Brown L, Brown RT, DuPaul G, Earls M, Feldman HM, Ganiats TG, Kaplanek B, Meyer B, Perrin J, Pierce K, Reiff M, Stein MT, Visser S (2011) ADHD: clinical practice guideline for the diagnosis, evaluation, and treatment of attention-deficit/hyperactivity disorder in children and adolescents. Pediatrics 128:1007–1022. 10.1542/peds.2011-2654 PMC4500647 22003063

[CIT0075] Wolraich ML , Hagan JF Jr, Allan C, Chan E, Davison D, Earls M, Evans SW, Flinn SK, Froehlich T, Frost J, Holbrook JR, Lehmann CU, Lessin HR, Okechukwu K, Pierce KL, Winner JD, Zurhellen W, Subcommittee On C, Adolescents With Attention-Deficit/Hyperactive Disorder (2019) Clinical Practice Guideline for the diagnosis, evaluation, and treatment of attention-deficit/hyperactivity disorder in children and adolescents. Pediatrics 144:e20192528. 31570648 10.1542/peds.2019-2528 PMC7067282

[CIT0076] Xu G , Strathearn L, Liu B, Yang B, Bao W (2018) Twenty-year trends in diagnosed attention-deficit/hyperactivity disorder among US children and adolescents, 1997-2016. JAMA Netw Open 1:e181471. 30646132 10.1001/jamanetworkopen.2018.1471 PMC6324288

[CIT0077] Yoshimasu K (2016) Substance-related and addictive disorders as a risk factor of suicide and homicide among patients with ADHD: a mini review. Curr Drug Abuse Rev 9:80–86. 27492359 10.2174/1874473709666160802112215

[CIT0078] Zwi M , Jones H, Thorgaard C, York A, Dennis JA (2011) Parent training interventions for attention deficit hyperactivity disorder (ADHD) in children aged 5 to 18 years. Cochrane Database Syst Rev CD003018. 22161373 10.1002/14651858.CD003018.pub3 PMC6544776

