# Supplementary material for: Exploring the Effects of Pharmacological, Psychosocial, and Alternative/Complementary Interventions in Children and Adolescents With Attention-Deficit/Hyperactivity Disorder: Meta-Regression Approach
Source: Int J Neuropsychopharmacol. 2021 Jun 4;24(10):776–86. doi: 10.1093/ijnp/pyab034 (PMC8538900; doi:10.1093/ijnp/pyab034)
Supplement: pyab034_suppl_Supplementary_Matrials [file pyab034_suppl_supplementary_matrials.docx]

**Table S1** Papers included in this meta-regression

| No. | Authors (Publication Year) | Title |
| --- | --- | --- |
| 1 | Coghill DR et al (2014) | Effects of Methylphenidate on Cognitive Functions in Children and Adolescents with Attention-Deficit/Hyperactivity Disorder: Evidence from a Systematic Review and a Meta-Analysis |
| 2 | Charach A et al (2013) | Interventions for Preschool Children at High Risk for ADHD: A Comparative Effectiveness Review |
| 3 | Maia CRM et al (2014) | Long-Term Efficacy of Methylphenidate Immediate-Release for the Treatment of Childhood ADHD: A Systematic Review and Meta-Analysis |
| 4 | Kelsey DK et al (2004) | Once-Daily Atomoxetine Treatment for Children with Attention-Deficit/Hyperactivity Disorder, Including an Assessment of Evening and Morning Behavior: A Double-Blind, Placebo-Controlled Trial |
| 5 | Huang Y.-H. et al (2015) | Treatment effects of combining social skill training and parent training in Taiwanese children with attention deficit hyperactivity disorder |
| 6 | Bangs ME et al (2008) | Atomoxetine for the Treatment of Attention-Deficit/Hyperactivity Disorder and Oppositional Defiant Disorder |
| 7 | Michelson D et al (2001) | Atomoxetine in the Treatment of Children and Adolescents with Attention-Deficit/Hyperactivity Disorder: A Randomized, Placebo-Controlled, Dose-Response Study |
| 8 | Handen BL et al (2015) | Atomoxetine, Parent Training, and Their Combination in Children With Autism Spectrum Disorder and Attention-Deficit/Hyperactivity Disorder |
| 9 | Reichow B et al (2013) | Systematic Review and Meta-Analysis of Pharmacological Treatment of the Symptoms of Attention-Deficit/Hyperactivity Disorder in Children with Pervasive Developmental Disorders |
| 10 | Hirota T et al (2014) | Alpha-2 Agonists for Attention-Deficit/Hyperactivity Disorder in Youth: A Systematic Review and Meta-Analysis of Monotherapy and Add-On Trials to Stimulant Therapy |
| 11 | Cortese S et at 2015) | Cognitive Training for Attention-Deficit/Hyperactivity Disorder: Meta-Analysis of Clinical and Neuropsychological Outcomes from Randomized Controlled Trials |
| 12 | Tang Y-N et al (2007) | The Efficacy of Atomoxetine in Treatment of Attention-Deficit Hyperactivity Disorder (ADHD): Meta-Analysis of RCT |
| 13 | Ghuman JK et al (2009) | Prospective, Naturalistic, Pilot Study of Open-Label Atomoxetine Treatment in Preschool Children with Attention-Deficit/Hyperactivity Disorder |
| 14 | Biederman J et al (2007) | Effect of comorbid symptoms of oppositional defiant disorder on responses to atomoxetine in children with ADHD: a meta-analysis of controlled clinical trial data |
| 15 | Newcorn JH et al (2006) | Low-Dose Atomoxetine for Maintenance Treatment of Attention-Deficit/Hyperactivity Disorder |
| 16 | Fan J et al (2011) | Effects of Methylphenidate Treatment on Parenting Stress in Children with Attention-Deficit/Hyperactivity Disorder |
| 17 | Gu J-W et al (2013) | Effect on social function of children with attention deficit hyperactivity disorder after methylphenidate-release treatment |
| 18 | Mohammadi MR et al (2016) | A Comparison of Effectiveness of Parent Behavioral Management Training and Methylphenidate on Reduction of Symptoms of Attention Deficit Hyperactivity Disorder |
| 19 | MTA Coop Group (1999) | A 14-Month Randomized Clinical Trial of Treatment Strategies for Attention-Deficit/Hyperactivity Disorder |
| 20 | Golubchik P. et al (2018) | Effectiveness of parental training, methylphenidate treatment, and their combination on academic achievements and behavior at school of children with attention-deficit hyperactivity disorder |
| 21 | Winters D.E. et al (2018) | Improvement in Irritability with Open-Label Methylphenidate Treatment in Youth with comorbid Attention-Deficit/Hyperactivity Disorder and Disruptive Mood Dysregulation Disorder |
| 22 | Yunhye Oh et al (2018) | Efficacy of Hippotherapy Versus Pharmacotherapy in Attention-Deficit/Hyperactivity Disorder: A Randomized Clinical Trial |
| 23 | Ghajar A et al (2018) | L-Carnosine as Adjunctive Therapy in Children and Adolescents with Attention-Deficit/Hyperactivity Disorder: A Randomized, Bouble-Blind, Placebo-Controled Clinical Trial |
| 24 | Gamli IS et al (2018) | Six months methylphenidate treatment improves emotion dysregulation in adolescents with attention/deficit hyperactivity disorder: a prospective study |
| 25 | Newcorn JH et al (2017) | Randomized, Double-Blind, Placebo-Controlled Acute Comparator Trials of Lisdexamfetamine and Extended-Release Methylphenidate in Adolescents with Attention-Deficit/Hyperactivity Disorder |
| 26 | Chen L-L et al (2007) | Clinical Research of Treating Children with Attention Deficit Hyperactivity with Fluoxetine Hydrochloride and Methylphenidate |
| 27 | Lin Y-L et al (2007) | Study on Combination Intervention for Attention Deficit Hyperactivity Disorder |
| 28 | Chen H-M et al (2008) | The Effect of Applying Cinnamon Aromatherapy for Children with Attention Deficit Hyperactivity Disorder |
| 29 | Jiang RH et al (2006) | Therapeutic Effect of Electroencephalograph Biofeedback on Cognitive Function of Children with Attention-Deficit Hyperactivity Disorder |
| 30 | Zhang YJ et al (2009) | Therapeutic Effect of Atomoxetine on Children with Attention Deficit Hyperactivity Disorder Comorbid Tic Disorder |
| 31 | Cao Q-J et al (2009) | Effects of Extended-Released Methylphenidate on the Inhibition of Children with Attention Deficit Hyperactivity Disorder |
| 32 | Wang Q-M et al (2010) | Effect of sandplay therapy in children with attention deficit hyperactivity disorders |
| 33 | Rejani TG et al (2012) | Efficacy of Multimodal Intervention for Children with Attention Deficit Hyperactivity Disorder (ADHD)—An Indian Study |

**Figure 1** Funnel plot

**Figure 2** The meta-regression plot of SMD as a function of the linear predicted values (Adjusted R^2^ = 35.22%); the circles are in proportion to the study weights in the meta-regression.
